# Somatic mutation signatures in primary liver tumors of workers exposed to ionizing radiation

**DOI:** 10.1038/s41598-019-54773-z

**Published:** 2019-12-03

**Authors:** David S. Goerlitz, Jan Blancato, Archana Ramesh, Md. Islam, Garrett T. Graham, Valentina Revina, Bhaskar Kallakury, Jay Zeck, Evgeniya Kirillova, Christopher A. Loffredo

**Affiliations:** 1 Lombardi Comprehensive Cancer Center, Georgetown University, Washington, District of Columbia USA; 2 Russian Radiobiology Human Tissue Repository, Southern Urals Biophysics Institute, Ozyorsk, Chelyabinsk Oblast Russian Federation; 3 Department of Pathology, Georgetown University, Washington, District of Columbia USA

**Keywords:** Cancer genomics, Cancer genomics

## Abstract

Liver cancer is associated with genetic mutations caused by environmental exposures, including occupational exposure to alpha radiation emitted by plutonium. We used whole exome sequencing (WES) to characterize somatic mutations in 3 histologically distinct primary liver tumors (angiosarcoma of the liver (ASL), cholangiocarcinoma (CCA) and hepatocellular carcinoma (HCC)) from Mayak worker subjects occupationally exposed to ionizing radiation (IR) to investigate the contribution of IR to the mutational landscape of liver cancer. DNA sequence analysis revealed these tumors harbor an excess of deletions, with a deletions:substitutions ratio similar to that previously reported in radiation-associated tumors. These tumors were also enriched for clustered mutations, a signature of radiation exposure. Multiple tumors displayed similarities in abrogated gene pathways including actin cytoskeletal signaling and DNA double-strand break (DSB) repair. WES identified novel candidate driver genes in ASL involved in angiogenesis and PIK3CA/AKT/mTOR signaling. We confirmed known driver genes of CCA, and identified candidate driver genes involved in chromatin remodeling. In HCC tumors we validated known driver genes, and identified novel putative driver genes involved in Wnt/β-catenin signaling, chromatin remodeling, PIK3CA/AKT/mTOR signaling, and angiogenesis. This pilot study identifies several novel candidate driver mutations that are likely to be caused by IR exposure, and provides the first data on the mutational landscape of liver cancer after IR exposure.

## Introduction

Epidemiological studies of atomic bomb survivors, nuclear workers and radiotherapy patients have shown that exposure to ionizing radiation is associated with increased lifelong risks for the development of solid tumors^1–3^. In particular, studies of the Mayak worker cohort strongly suggest that human occupational exposure to ionizing radiation from plutonium (isotope ^239^Pu) used to manufacture nuclear weapons is associated with increased risks for lung, liver and bone cancer, the primary tissues of plutonium deposition after exposure, and the organs receiving the largest doses^4,5^.

The Mayak worker cohort consists of 22,373 workers of both genders employed at the Mayak Production Association Facility, which was built by the Soviet Union in 1948 in the southern Ural Mountains region (Ozyorsk, Chelyabinsk Oblast) to produce nuclear material for the Russian weapons program^6^. Radiochemical and plutonium production plant workers were occupationally exposed to both internal (alpha) radiation and external (gamma) sources with a wide range of exposures^7^. During its first decade of operations (1948–1958), Mayak workers were exposed to high levels of inhaled or ingested ^239^Pu^8,9^. Workers in the radiochemical plant were exposed primarily to plutonium nitrate (^239^Pu (NO_3_)_4_), while plutonium production facility workers were exposed to plutonium oxide (^239^PuO_2_)^10^. While improved occupational hygiene practices implemented in 1958 reduced exposure overall, workers still experienced chronic low-dose exposure until primary weapons production ceased in 1982. Therefore, this cohort provides a unique resource to study human carcinogenic risks of chronic plutonium exposure to both external gamma rays and internal alpha radiation.

Epidemiological studies of the Mayak worker cohort provide direct evidence that occupational levels of plutonium exposure increase liver cancer risk in humans. The excess relative risk (ERR) for liver cancer among both male and female Mayak workers with internal plutonium exposures was shown to be significantly increased with increasing cumulative internal liver dose (ERR = 4.9, CI = 2.0–12.2, p < 0.001)^5^. Furthermore, association with plutonium dose was significantly modified by gender: ERRs for liver cancer were estimated at 2.6 and 29 for males and females, respectively^4^. Of 46 histologically confirmed liver tumors in the Mayak cohort that were found to be associated with internal plutonium exposure, hepatocellular carcinoma (HCC) was the most frequently (59%) observed type, followed by angiosarcoma of the liver (ASL) (22%) and cholangiocarcinoma (CCA) (17%)^5^. ASL tumors were observed only at high (≥4 Gy) internal plutonium doses while CCA cases were observed only in workers with less than 0.5 Gy liver dose^5^. No statistically significant association was detected between external gamma-ray dose and liver cancer risk in this cohort^4,5^. To the best of our knowledge, the collection of stored tissues from the Mayak cohort represents the largest case series of workers with liver cancer following plutonium exposure.

ASL is a rare vascular tumor arising from endothelial cells of blood or lymphatic vessels and accounts for only 2% of primary liver malignancies^11^. The annual incidence of ASL is estimated at one confirmed case per 25 million people^12^. However, the annual incidence of ASL in the Mayak worker cohort is estimated at 1.3 cases per 100,000 people, a 250x increase^5^. The most common risk factor for the development of ASL is therapeutic radiation^13^, which was particularly well documented after exposure to the thorium containing contrast agent Thorotrast during its use from 1930–1960^14^. HCC is a malignancy arising from hepatocytes, and is the most common type of primary liver cancer representing 70–90% of all cases^15^. The major risk factors for HCC are well known and include hepatitis B (HBV) and C (HCV) viral infections, alcohol abuse, and exposure to aflatoxin B1^16^. Intrahepatic CCA develops in the cells of the small bile ducts within the liver. CCA is the second most common primary liver cancer, accounting for 10–20% of cases^17^. Its main risk factors include HBV and HCV infections, chronic inflammation from liver fluke infestation, primary sclerosing cholangitis, and inflammatory bowel disease^18^.

The study of occupational exposures to radiation in the Mayak cohort provides the ability to generate unique data about the influence of radiation-induced DNA mutations in primary liver cancer. Current dogma follows the paradigm that liver tumors produced through different environmental carcinogen exposures have somatic DNA mutations that influence specific target genes, and as a consequence, produce changes driving different carcinogenic pathways*.* Despite the strong epidemiological evidence of increased liver cancer risk in exposed individuals in this cohort, little is known about the mutational landscape of these tumors. As all subjects in this study were occupationally exposed to ionizing radiation, the primary liver tumors analyzed here represent unique biospecimens to investigate the potential contribution of ionizing radiation exposure to the mutational spectrum of liver tumor genomes. The goal of this study was to use whole exome sequencing to characterize somatic mutations in histologically distinct primary liver tumors (ASL, CCA and HCC) from Mayak worker cohort subjects to identify potential driver mutations and signature mutational events, which may reveal underlying genes and pathways driving liver tumorigenesis. This, in turn could suggest new targets for therapeutic intervention, and facilitate the development of biomarkers to screen populations with high risks of liver cancer.

## Results

### Clinicopathological characteristics of subjects with primary liver tumors

**Figure 1:**
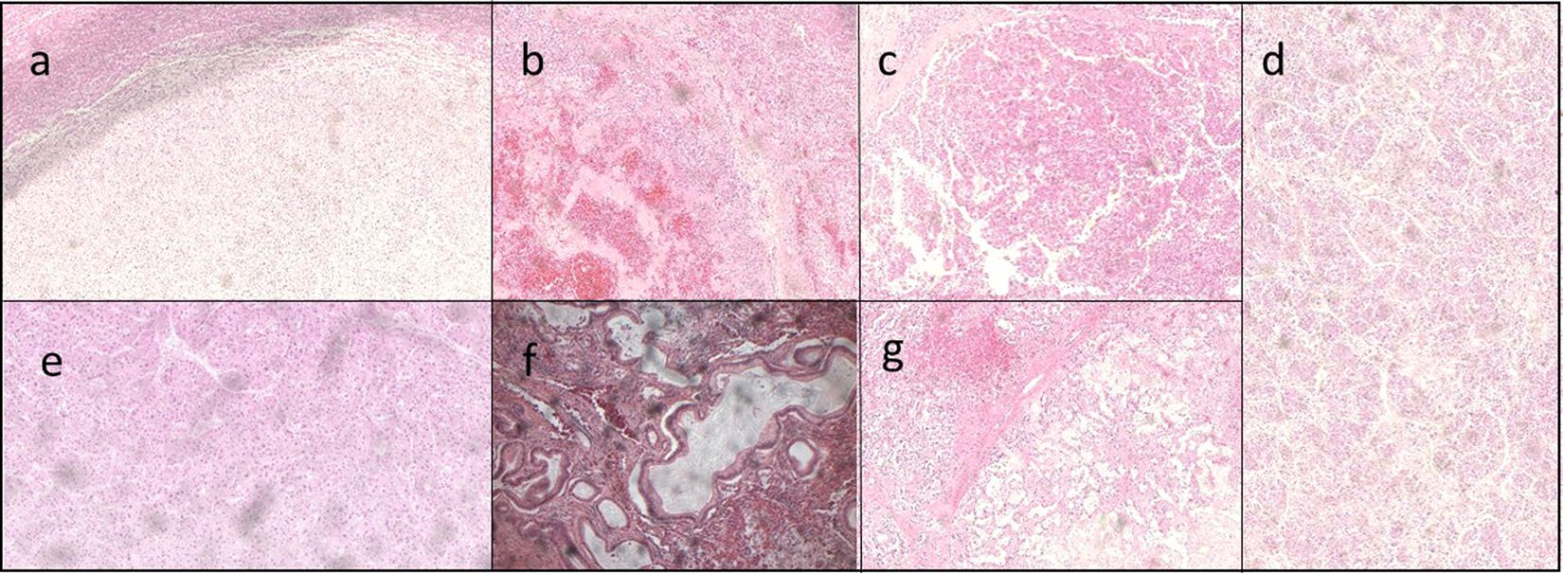
Representative histological images. (**a**) ASL_116549: Hypercellular spindle cell proliferation without evidence of necrosis at the interface of hepatic parenchyma, consistent with angiosarcoma. (**b**) ASL_116551: Prominent vascular proliferation showing distended vessels lined by atypical cells as noted in a background of hepatic tissue, consistent with angiosarcoma. (**c**) HCC_116565: Markedly atypical cells with enlarged nuclei and prominent nucleoli, arranged in nests and showing abundant eosinophilic cytoplasm, consistent with hepatocellular carcinoma. (**d**) HCC_116569: Neoplastic cells with enlarged nuclei seen in a nested pattern with eosinophilic cytoplasm in a background of benign hepatic parenchyma, consistent with hepatocellular carcinoma. (**e**) HCC_116577: Atypical hepatocytes showing some nuclear enlargement and a nested architecture without evidence of portal tract areas and with notable neovascularization, consistent with a well-differentiated hepatocellular carcinoma. (**f**) CCA_116583: Haphazardly arranged neoplastic glands, consistent with cholangiocarcinoma. (**g**) CCA_116595: Haphazardly arranged neoplastic glands, consistent with cholangiocarcinoma.

The seven cases of primary liver cancer were angiosarcoma of the liver (ASL; n = 2), cholangiocarcinoma (CCA; n = 2), and hepatocellular carcinoma (HCC; n = 3). Representative histology of tumors revealed typical morphology of each respective tumor type as shown in Fig. 1. Patient demographics, including doses of radiation are detailed in Supplemental Table 1.

### Mutation spectrum identified from primary tumors

To comprehensively characterize somatic mutations in primary liver tumors from subjects occupationally exposed to ionizing radiation, we analyzed WES data from 14 FFPE tissue samples of paired primary liver tumor and adjacent non-tumor tissue from seven subjects. The average sequencing depth of coverage was 780x for tumor samples and 690x for paired adjacent non-tumor samples.

The mean number of nonsynonymous mutations per subject was 380.4 (range 24–857). Across all seven tumors, we identified a mutation spectrum dominated by C:G > T:A transitions, as shown in Supplemental Table 2, which is comparable to the predominant somatic mutations found in ASL^19^, CCA^20^, HCC^21^, and other cancers^22^. An average of 4.9 (range 0.35–11.24) nonsynonymous SNVs/Mb sequenced, and 0.8 (range 0.02–1.74) indels/Mb are shown in Supplemental Table 3.

An excess of deletions is a signature of ionizing radiation exposure in radiation-associated vs radiation-naïve malignancies irrespective of tumor type^23^. We found a deletion:substitution ratio = 0.072 in these tumors, which is comparable to the ratio = 0.082 previously reported in radiation-associated second malignancies^23^. As deletions can be caused by alignment errors in the context of short tandem repeats, we reviewed the sequence motifs of the flanking sequences and breakpoints of all deletions to detect short tandem repeats. In total, of 553 deletions detected, only 5 (<1%) were found close to short tandem repeats. The sequence alignments in this study were additionally processed using GATK (v3.3-0) to recalibrate base qualities and correct alignment errors and artifacts that arise due to indels. Tumor-normal pairs were also co-realigned for the somatic variant analysis to ensure that the indel positions are consistent between the matched samples.

We found the frequency of clustered mutations (1–3 SNVs within 5 base-pairs of each other) to average 8.6% across seven tumors (range 6.0–12.1%), higher than the frequency of 1.5% reported as significantly enriched in the offspring of irradiated males compared with controls in mice^24^. Clustered mutations are a signature of radiation exposure^25,26^. These data further support the explanation that the mutations described here in these primary liver tumors may be induced by exposure to ionizing radiation.

Three tumor samples in this study exceeded the somatic mutation burden of 5.0 SNVs/Mb classified as hypermutated in previous studies of HCC and CCA, where the cohort included several cases that harbored nonsynonymous mutations in genes involved in DNA mismatch repair, DNA polymerase, or nucleotide excision repair^21,27^. We identified nonsynonymous mutations in nucleotide excision repair (*ERCC3* and *XAB2*), DNA polymerase subunit (*POLE, POLQ*, and *REV3L*), homologous recombination (*BRCA1, BRCA2*, and *ATM*), and non-homologous end-joining (*LIG4*) genes in 5/7 (71%) liver tumors in this study, suggesting a lack of correlation between DNA repair capacity and somatic mutation rate. Previous data from a report describing mutational signatures of ionizing radiation exposure suggests that repair of radiation-induced DNA damage may occur primarily through microhomology mediated or non-homologous end-joining pathways, rather than through homologous recombination^23^.

### Genetic alterations in primary liver tumors

**Figure 2:**
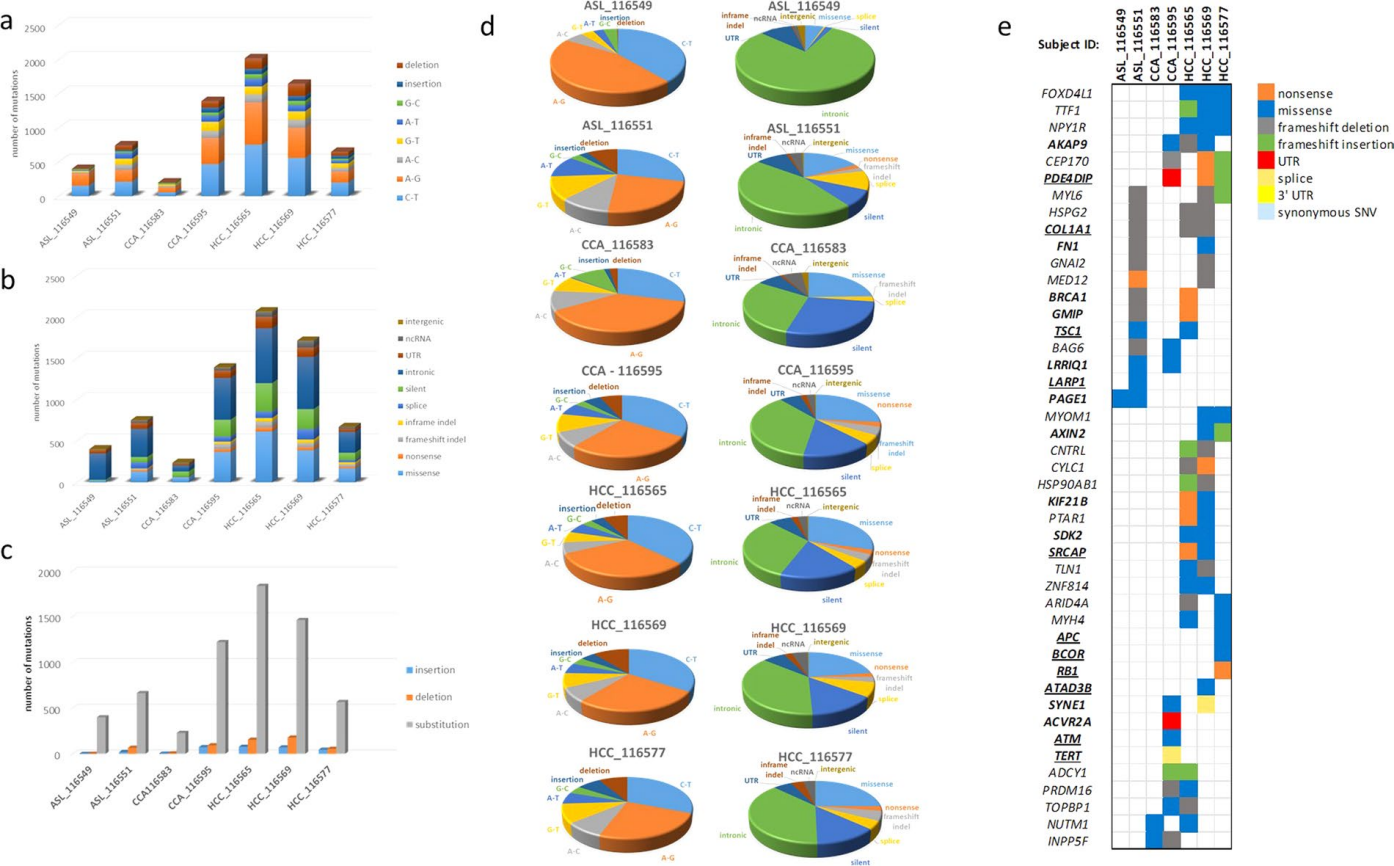
Somatic mutation spectrum in primary liver tumors. Summary of tumors sequenced and the frequencies of mutations seen. (**a**) Frequencies of specific types of substitutions by mutation class for each individual tumor (**b**) Frequencies of specific types of substitutions by translation effect for each individual tumor (**c**) Frequencies of insertions, deletions and SNVs. (**d**) Pie charts showing the distribution of mutations by type of alteration and translation effect (**e**) Co-occurrence of mutations in each tumor sequenced. Recurrently mutated genes are shown on the y-axis and subject IDs on the x-axis. The matrix in the center of the figure represents individual mutations in each tumor. Genes in bold are previously reported in the literature for HCC, CCA and/or ASL; genes underlined are determined as significantly mutated, putative driver genes in previous reports. For each panel the alteration types were represented by different colors, respectively.

Candidate driver gene identification was based primarily on recurrence of the same altered gene in multiple tumors (Fig. 2), and the functional impact of mutations (SIFT, PolyPhen, and Ensembl Variant Effect Predictor). In addition, the presence of the gene in the COSMIC database and existing evidence supporting the role of the gene as a cancer driver gene in ASL, CCA or HCC were considered. Additional singleton mutations of interest are listed in Supplemental Table 4.

In one ASL and two HCC tumors, we discovered recurrent frameshift deletions in *HSPG2* (Heparan Sulfate Proteoglycan 2) which encodes the perlecan protein, a key component of the vascular extracellular matrix, which modulates inhibition of angiogenesis by *TSP1* (Thrombospondin 1)^28^. To our knowledge, mutation of *HSPG2* has not been previously reported in ASL or HCC. PIK3CA/AKT/mTOR pathway gene *TSC1* (Tuberous Sclerosis 1), a known cancer driver gene in HCC^21^ was recurrently altered via missense mutation in one ASL and one HCC tumor in this cohort. Alteration of *TSC1* has not been previously reported in ASL. In addition, we identified recurrent mutations in two genes involved in actomyosin dynamics in ASL and HCC: *COL1A1* (Collagen Type I Alpha 1 Chain) and *GMIP* (GEM Interacting Protein), both of which have been previously reported as mutated in HCC^29–31^, but have not been previously described in ASL. *COL1A1* is recurrently altered via frameshift deletion in one ASL and two HCC tumors in this cohort. *GMIP* is altered via frameshift deletion in one ASL tumor, and by nonsense mutation in one HCC tumor. Altered genes abrogating other signaling pathways in ASL tumors in this cohort include Wnt/β-catenin signaling gene *MED12* (Mediator Complex Subunit 12), and DNA damage response gene *BRCA1*. Both *MED12* and *BRCA2* were mutated in one ASL and one HCC tumor. Although *BRCA1* is involved in DNA damage response and has been reported in CCA^32,33^, this is the first report of mutation of this gene in ASL.

We identified recurrent mutations in two genes involved in chromatin remodeling in CCA tumors in this cohort: *NUTM1* (NUT Midline Carcinoma Family Member 1) and *PRDM16* (PR/SET Domain 16). *NUTM1* mutations were found in both CCA and HCC tumors. In one CCA tumor and two HCC tumors we identified recurrent mutations in *PDE4DIP* (Phosphodiesterase 4D Interacting Protein), a gene involved in cAMP signaling and a known driver gene in HCC^34^. Only 1 gene, *INPP5F* (Inositol Polyphosphate-5-Phosphatase F), a modulator of the AKT-STAT3 signaling pathway, was found to be mutated in both CCA tumors in this study, a novel finding.

Mutations in Wnt/β-catenin signaling pathway member genes *AXIN2* (Axin 2), and *MED12* (Mediator Complex Subunit 12) were present in multiple HCC tumors. Additional genes involved in chromatin remodeling were also recurrently mutated in HCC, including *SRCAP* (Snf2 Related CREBBP Activator Protein), *TTF1* (Transcription Termination Factor 1), and *ARID4A* (AT-Rich Interaction Domain 4A). Two HCC tumors harbored mutations in the *SRCAP* gene, a keycomponent involved inchromatin-remodeling. Inactivating frameshift and nonsense mutations in *SRCAP* have been previously reported in HCC^21^, suggesting a tumor-suppressor function in HCC. *TTF1* encodes a transcription termination factor, and frameshift deletions and missense mutations in *TTF1* co-occur in all 3 HCC tumors in this cohort. We found *ARID4A* to be recurrently mutated by a frameshift deletion and a nonsense mutation in two HCC samples. While *ARID4A* is not described in COSMIC or previous sequencing studies of liver cancer, recurrent mutations in the paralogous nucleosome remodelers *ARID1A, ARID1B*, and *ARID2* have been previously reported in HCC^21,30,35^. In concordance with these previous data, the mutations in *ARID* genes identified in this study are predicted to result in protein inactivation, further establishing *ARID* genes as potential HCC tumor suppressors.

### Mutation signature analysis

**Figure 3:**
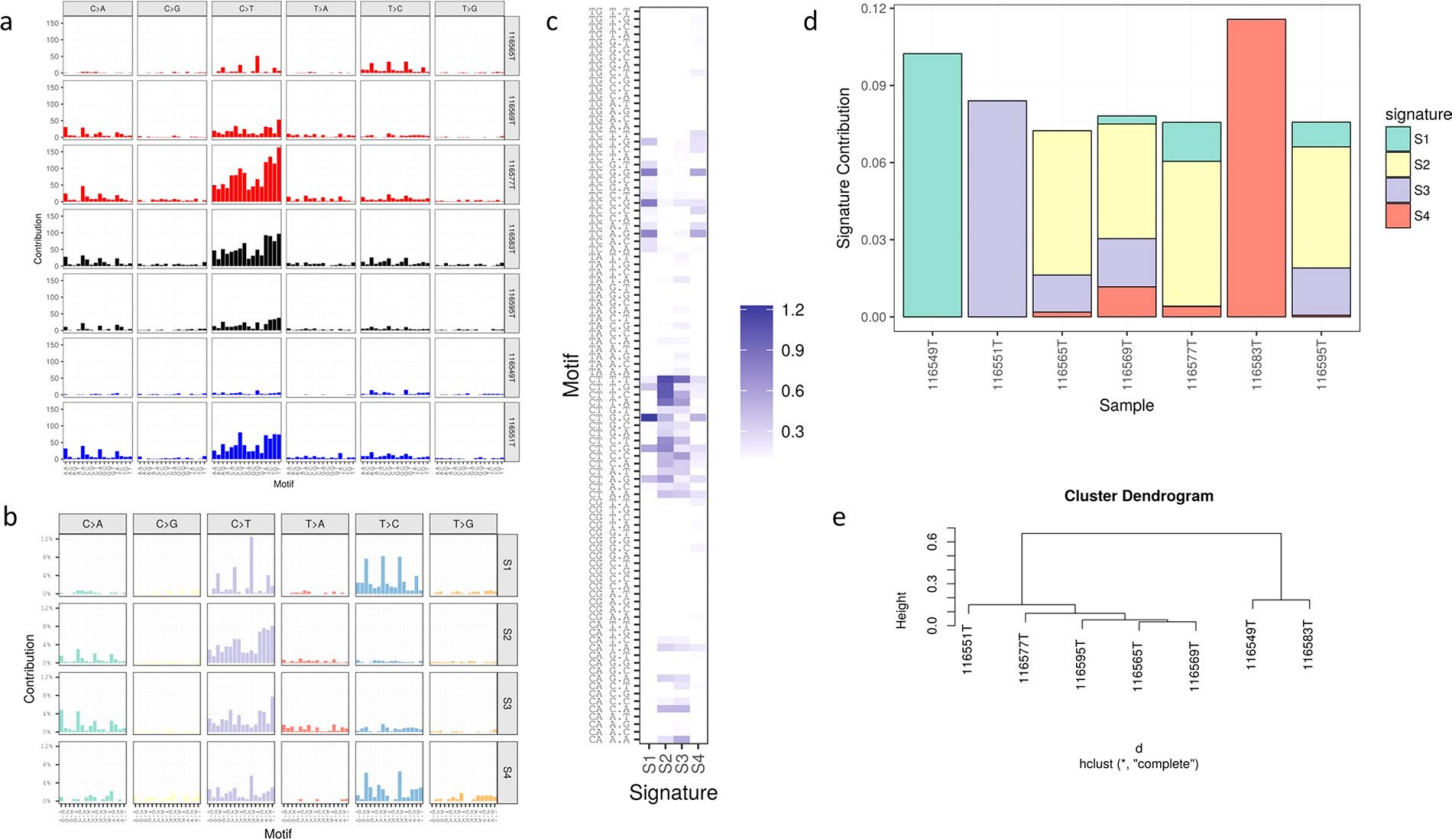
Analysis of mutational signatures for 7 primary liver tumors. The observed mutational spectrum of each tumor (**a**) was decomposed into four distinct mutational signatures S1-S4 (**b**). (**c**) Composition of somatic signatures S1-S4 estimated by non-negative matrix factorization (NMF), represented as a heatmap. (**d**) Contribution of the four mutational signatures to each tumor. (**e**) Hierchical clustering of the mutational spectrum by tumor.

To further explore trinucleotide-context mutation spectra in tumors in this cohort, we usednon-negative matrix factorization to extract four stable and reproducible mutational signatures as shown in Fig. 3. We compared these signatures to those from the Catalogue of Somatic Mutations in Cancer (COSMIC) (http://cancer.sanger.ac.uk/cosmic/signatures). We identified similarity to a number of established mutation signatures. These included COSMIC signatures associated with microsatellite instability (Signature 21), and high numbers of indels and defective DNA mismatch repair (Signature 26).

### Pathway analysis

**Figure 4:**
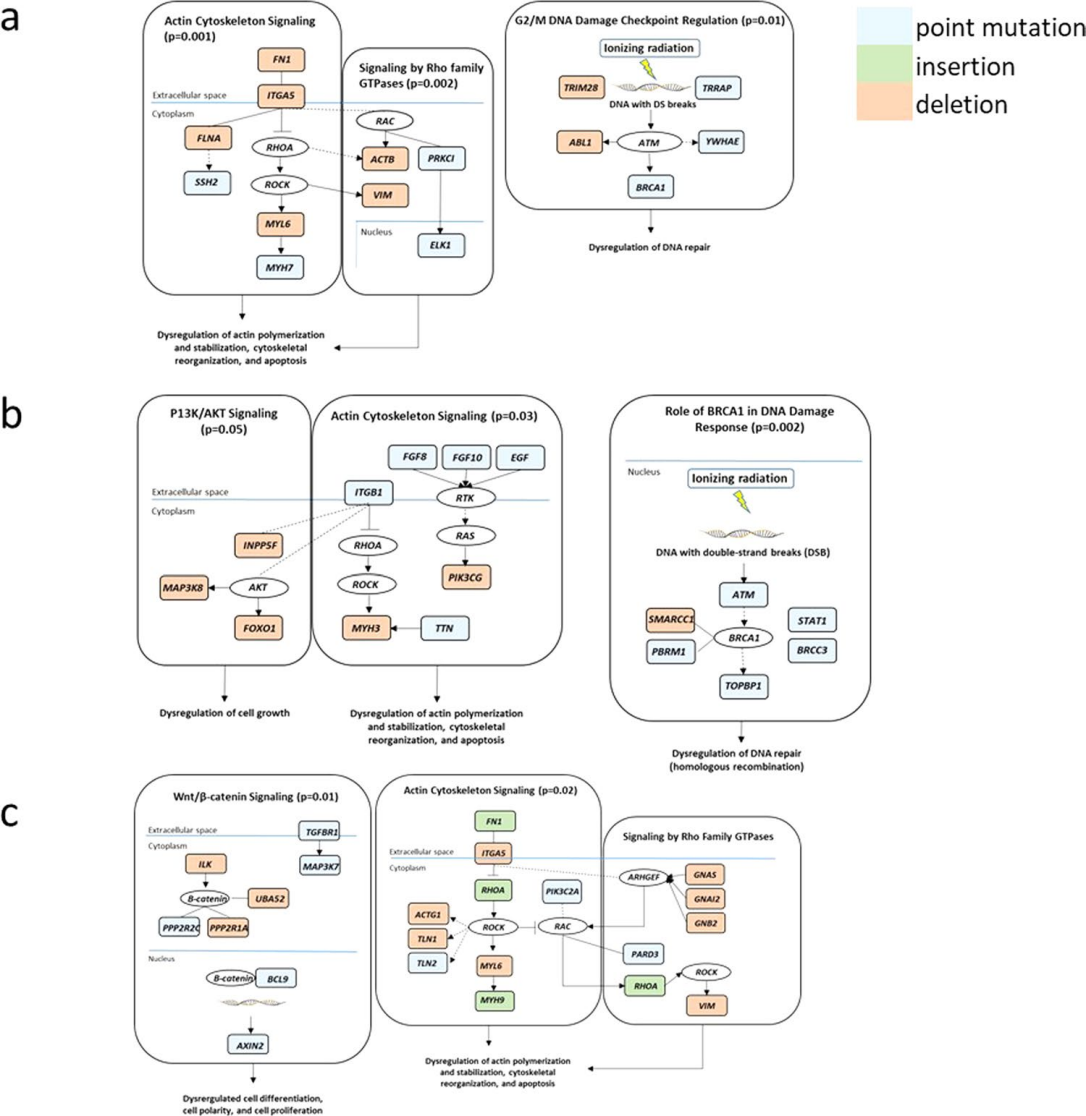
Altered cancer pathways in ASL, CCA and HCC. Core pathway analysis identified genomic alterations in multiple canonical pathways. Alterations in (**a**) ASL, (**b**) CCA, and (**c**) HCC include point mutations, insertions and deletions. Alteration types were represented by different colors, respectively. P-values represent significance (p = 0.05, Fisher’s exact test; not significant under FDR or Bonferroni correction).

Five of seven tumors were significantly (p = 0.05, Fisher’s exact test; not significant under FDR or Bonferroni correction) enriched for genetic alterations in the canonical actin cytoskeletal signaling pathway, and DNA double-strand Break (DSB) repair pathways (Fig. 4). Enrichment of mutations in genes involved in cell assembly, organization, andmorphology are suggestive of *in vivo* exposure to ionizing radiation^23,36^.

## Discussion

**Figure 5:**
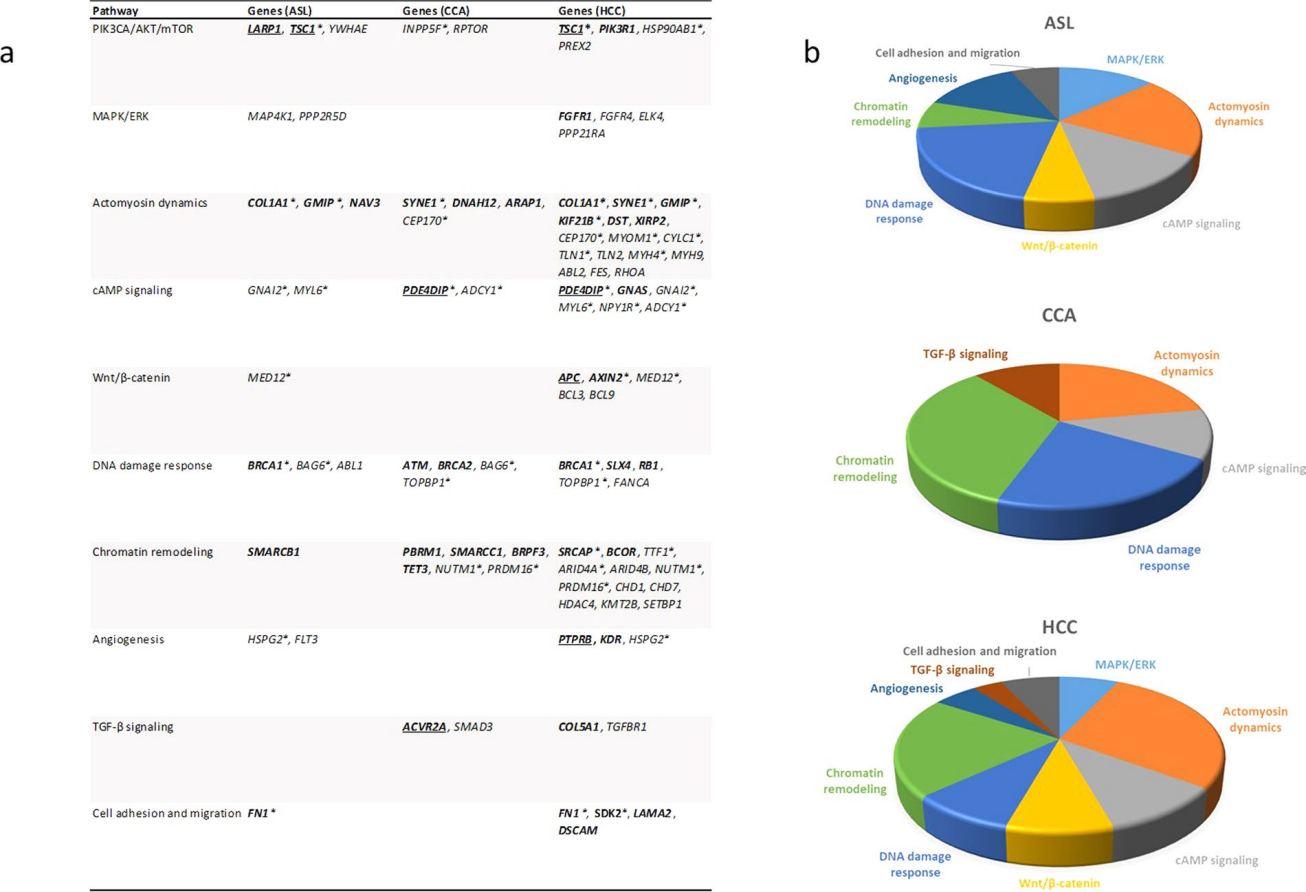
Classification of select genomic alterations identified by roles in cell signaling pathways. (**a**) Genes in bold are previously reported in the literature for HCC, CCA and/or ASL; genes underlined are determined as significantly mutated, putative driver genes in previous reports; asterix indicate recurrently mutated genes (**b**) Pie charts depicting the frequency of the number of genes mutated in a given biological pathway for each tumor type.

Our data are consistent with previous studies, which describe a complex mutational landscape of ASL, CCA and HCC. The small number of tumors analyzed in this study, coupled with considerable genetic heterogeneity, resulted in a general lack of strong evidence for specific driver mutations. However, our analysis did validate a number of known driver genes in both CCA and HCC, while revealing a number of novel candidate drivers in ASL, CCA and HCC (Fig. 5). We describe here that these tumors harbor an excess of deletions and clustered mutations, both of which are considered genetic signatures of exposure to ionizing radiation.

After cellular exposure to ionizing radiation (IR), the first step in DNA damage response is detection of DNA damage by ataxia-telangiectasia mutated protein (ATM) and ataxia-telangiectasia and Rad-3 related protein (ATR). ATM is the master regulator monitoring the genome and responding to double-stranded breaks (DSB) induced by exposure to IR^37^, while ATR is typically activated after detection of single-stranded breaks (SSB) and stalled replication forks^38^. Both ATM and ATR are nucleus-localized kinases, which function to phosphorylate and activate various downstream proteins involved in cell cycle arrest. These target proteins including TP53, and repair enzymes Rad51, Chk1 and Chk2^39,40^. ATM and ATR both respond to radiation-induced DNA damage by phosphorylating BRCA1, thus activating the protein to assist in homologous recombination repair. *BRCA1* was mutated via frameshift deletion in one ASL tumor and via nonsense mutation in one HCC tumor in this cohort, representing what we believe to be the first known reports of mutation of this gene in ASL and HCC. Mutations in *BRCA1* have been previously reported in CCA^32,41^. These *BRCA1* mutation data suggest a role for radiation-induced pathogenesis in both ASL and HCC tumors in this cohort.

It is worth noting that the only recurrently mutated gene in both ASL tumors in this cohort is *PAGE1*, which encodes a protein about which nothing is presently known about its function. Interestingly, *PAGE1* was previously identified as one of seven genes included in a radiation-induced gene signature that completely distinguished post-Chernobyl papillary thyroid cancers (PTC) from non-radiation induced PTC^42^. MutSigCV analysis revealed this gene mutation to be significant (p = 9.42 × 10^−4^), but it did not meet significance with FDR correction (q = 1).

Recent studies^27,33,43^ using next-generation sequencing have revealed that the mutational landscape of CCA differs significantly by etiology. However, a series of highly recurrent mutations in genes associated with chromatin remodeling (*PBRM1* and *ARID1A*), PIK3CA/AKT/mTOR pathways (*IDH1* and *IDH2*), and cell cycle control (*TP53, KRAS* and *BRAF*) appear to be common pathways in cholagiocarcinogenesis. In the CCA tumors in the Mayak cohort, we identified mutations in multiple genes involved in chromatin remodeling, including two recurrently mutated genes described in COSMIC but not previously reported in primary liver tumors: *NUTM1* and *PRDM16*. *NUTM1* is associated with Nut Midline Carcinoma (NMC), a squamous cell epithelial cancer characterized by a *BRD-NUT* translocation involving the rearrangement of *BRD4* (bromodomain-containing protein 4) and *NUTM1*^44^. The putative role of oncogenic missense mutations in *NUTM1* in CCA pathogeneisis is unclear, however a recent report supports a model wherehistone hyperacetylation initiates transcriptional activation, which underlies oncogenesis in NMC^44^. The protein encoded by the *PRDM16* gene is a zinc finger protein, which binds DNA and functions as a regulator of chromatin remodeling and transcriptional activation. A recent study using RNA interference screening to interrogate the function of histone methyltransferases and demethylases in prostate cancer cells identified *PRDM16* to have a role in allowing cells to evade apoptosis^45^. In addition, the *PRDM16* gene is described as oncogenic in acute myelocytic leukemia^46^. Previous reports demonstrate the alteration of at least one chromatin-remodeling gene in 47% of CCA cases^47^. Our data from CCA tumors in this cohort reinforce the key role of epigenetic modification in this malignancy.

The only gene found to be mutated in both CCA tumors in our study is *INPP5F*, a modulator of the AKT-STAT3 signaling pathway. This gene was inactivated via deletion and missense mutation in both CCA tumors, consistent with the observation that deregulation of the P13K/AKT/mTOR pathway is a hallmark of intrahepatic CCA. The *INPP5F* gene product negatively regulates the STAT3 signaling pathway by inhibiting phosphorylation of STAT3 but the functional role of the gene in primary liver tumor pathogenesis has not been characterized. To our knowledge, *INPP5F* has not been previously reported in sequencing studies of CCA. A recent report suggests that *INPP5F* acts as a tumor suppressor in gliomas through a mechanism involving STAT3 pathway inhibition^48^. INPP5F also functions as a protein tyrosine phosphatase (PTP) in the P13K/AKT/mTOR pathway. Radiation exposure can induce pro-survival signaling pathways (e.g. P13K/AKT/mTOR), resulting in evasion of apoptosis, and initiation of cell cycle arrest and/or DNA-repair^49,50^. An increase in HER1-4 phosphorylation and subsequent activation following ionizing radiation exposure has been reported in breast cancer cells^51^, potentially through the mechanism of reactive oxygen/nitrogen species (ROS/RNS)-induced inhibition of PTPs, which suppress HER receptor tyrosine kinase (RTK) phosphorylation of HER^52,53^. Thus, ionizing radiation exposure could potentially increase radiation resistance in tumor cells through the mechanism of HER receptor activation of P13K/AKT/mTOR signaling, enhanced by PTP suppression, which plays a critical role in suppression of apoptosis.

Similar to other solid tumors, HCC is a genetically heterogeneous malignancy. Each individual tumor is comprised of a unique repertoire of somatic genetic alterations resulting from the accumulation of mutations influenced by distinct etiologies and environmental exposures. *TERT* activation via promoter point mutations, HBV integration, or focal amplifications is the most frequently mutated gene in HCC^30^ but these were not seen in this study. After *TERT*, the most recurrently mutated (~30%) gene in HCC is *CTNNB1* (Catenin Beta 1), which codes for β-catenin, a protein which acts as a key intracellular signal transducer in the canonical Wnt/β-catenin signaling pathway^21,29–31,54,55^. Dysregulation of the Wnt/β-catenin signaling pathway, which plays a role in cell survival, proliferation, migration and invasion, is an early event in hepatocarcinogenesis and has been associated with an aggressive HCC phenotype. We did not identify any mutations in *CTNNB1* in any of three HCC tumors in this study, however we did identify recurrent nonsynonymous mutations in two genes involved in the Wnt/β-catenin signaling pathway: *AXIN2* and *MED12*.

Genes involved in the SWI/SNF (SWItch/Sucrose Non-Fermentable) nucleosome-remodeling complex (*SRCAP* and *ARID4A*) and other chromatin remodeling genes (*TTF1, NUTM1*, and *PRDM16*) were recurrently mutated in these HCC tumors. Snf2 Related CREBBP Activator Protein gene *SRCAP*, involved in histone modification, is a known driver gene in HCC^21,30^. *TTF1* encodes a multifunctional nucleolar transcription termination factor that plays a critical role in the termination of ribosomal gene transcription. Interestingly, while *TTF1* is not described in any previous sequencing studies of liver cancer, it was altered in all three HCC tumors in this study. We found *ARID4A* to be recurrently mutated by a frameshift deletion and a nonsense mutation in two HCC samples. Recurrent mutations in the paralogous nucleosome remodelers *ARID1A, ARID1B*, and *ARID2* have been previously reported in HCC^21,30,35^. In concordance with these previous data, the mutations in *ARID* genes identified in this study are predicted to inactivate protein function, further establishing *ARID* genes as potential HCC tumor suppressors.

While artificial base alterations (e.g. C > T and G > A) have been detected in low-confidence mutation calls obtained from FFPE samples, high-confidence mutation calls in FFPE samples have been shown to be compatible to that in matched frozen samples^56^. The sequence datasets presented in this study underwent stringent criteria to optimize sensitivity while ensuring to filter out false-positives potentially associated with FFPE samples. For example, a minimum sequencing depth of 80x is recommended to obtain high-confidence NGS data from FFPE^57^, therefore in this study we increased the average sequencing depth of coverage to 780x for tumor samples and 690x for paired adjacent non-tumor samples. In addition, the Agilent SureSelectXT Human All Exon library preparation kit chemistry and protocol used in this study has been previously assessed for performance using low quality FFPE-derived DNA from lung adenocarcinoma samples^58^. A comparison of WES data of gDNA isolated from 5 fresh frozen (FF) and 5 matched FFPE samples found sequencing metrics were optimal and comparable among FFPE and FF samples^58^. Using the same analytical tools used in this study (BWA-MEM, Picard, GATK HaplotypeCaller), their comparison of single nucleotide variants (SNVs) between matched FFPE-FF pairs revealed a percentage of shared SNVs ≥ 90%^58^. In addition, a genotype concordance rate ≥ 97% between matched FFPE-FF pairs was calculated at different coverage thresholds (10 × −50 × ), and they concluded that high-quality, reproducible data can beobtained from WES of FFPE samples^58^.

The sequence analysis tools used in this study are optimized for FFPE and produce highly refined variant calls based on variant quality score recalibration (VQSR), wherein low confidence and likely false positive variants are filtered by applying thresholds to mapping quality, normalized variant quality, and other variant statistics that indicate the presence of strand bias, differences in mapping qualities, and position of ref/alt bases in reads supporting the reference and alternate alleles. In this study, we went further and concurrently analyzed high confidence mutation call sets from each tumor and matched adjacent non-tumor sample to detect somatic tumor-specific single nucleotide substitutions and small indels (<100 bp) using a combination of MuTect and VarScan. MuTect and VarScan are both industry-standard tools that apply multiple filters to detect false positive mutationsdue to errors in mapping and/or sequencing. In addition, llele frequency thresholds were applied to variants to identify them as high-confidence (e.g. variant allele frequency >5% in tumor and <5% in normal).

To the best of our knowledge, this pilot study is the first report of WES analysis of histologically distinct liver tumors from a cohort occupationally exposed to ionizing radiation. In addition to well-known cancer genes, our study identified several recurrent mutations in ASL, CCA and HCC in genes whose functional roles in cancer are currently unknown. However, the mutational evidence reported here warrants further investigation and/or validation. DNA sequence analysis revealed these tumors harbor an excess of deletions, with a deletions:substitutions ratio similar to that previously reported in radiation-associated tumors. In addition, we found the frequency of clustered mutations, a signature of radiation exposure, to be higher in these tumors than previously reported in irradiated mice. Multiple tumors displayed similarities in abrogated gene pathways including actin cytoskeletal signaling and DNA double-strand break (DSB) repair. In addition, WES identified several novel candidate driver genes involved in angiogenesis, PIK3CA/AKT/mTOR signaling, Wnt/β-catenin signaling, chromatin remodeling, cAMP signaling, and immune responses. Future studies will involve analysis of additional samples by next generation sequencing and mRNA analysis to replicate and validate our pilot study findings, and will include tumor tissue from residency-matched controls not exposed to ionizing radiation. In addition, as recent evidence suggests that *in vivo* exposure to ionizing radiation results in recurrent chromosomal alterations, including significant increases in balanced inversions, aCGH or whole genome sequencing will provide greater resolution to interrogate genetic signatures of IR exposure. This study also demonstrates the great potential of the biospecimens housed in the RHTR to be used for future experiments using cutting edge genomic technologies to answer seminal questions on the genetic landscape of IR-induced tumorigenesis.

## Methods

### Specimen selection

Formalin-fixed paraffin embedded (FFPE) primary tumors with adjacent non-tumor tissues were available from 32 of 46 Mayak workers in the cohort who developed liver cancer. The specimens were obtained at autopsy during the period of 1978–1990, stored in the Russian Radiobiology Human Tissue Repository (RHTR) of the Southern Urals Biophysics Institute (SUBI) in Ozyorsk, Russia, and shipped to Georgetown University Medical Center (GUMC). For this pilot study we selected seven cases (three HCC, two CCA, and two ASL) whose slides contained the maximal amount of tumor tissue of the highest histological quality to ensure a high yield of DNA, and sufficient area of adjacent non-tumor tissue to allow DNA extraction for comparison to tumor DNA. All workers were occupationally exposed to ionizing radiation and monitored for external gamma exposure (Gy) using individual film badges^5^, and these data are available for all seven subjects in this study. Measures of internal ^239^Pu does were estimated from plutonium concentrations in urine and calculated using biokinetic models^59^. Tumor samples and annotated medical records were anonymized. Informed consent for research was obtained by RHTR personnel from donors or next-of-kin, and the protocol was approved by SUBI’s Institutional Review Board. The Georgetown University Institutional Review Board approved an exemption request to study the de-identified materials.

### Pathological examination of tumor and adjacent non-tumor samples

FFPEs were sectioned, and hematoxylin & eosin (H&E) stained slides were generated in the GUMC Histopathology and Tissue Shared Resource. The slides had been previously reviewed by surgical pathology and recorded at the RHTR (VR), and independently confirmed by a Clinical Pathologist (BK) at GUMC. The diagnosis was recorded, together with tumor grade, percentage of visible tumor, non-tumor, necrotic tissues, and evidence of cirrhosis and chronic hepatitis. Representative images of the H&E slides were taken using the Olympus IX-71 imaging microscope at the GUMC Microscopy and Imaging Shared Resource.

### DNA isolation

For each case, serial 10 µM sections were cut from FFPE blocks, and areas with tumor and adjacent non-tumor tissue were macrodissected from slides using the H&E slides as templates. Genomic DNA was extracted using proteinase K digestion, phase-separation with phenol:chloroform:isopropol alcohol, and ethanol precipitation. The DNA quality and quantity were analyzed with UV-VIS using the NanoDrop spectrophotometer (Thermo Fisher), and fluorometry using the Qubit 2.0 Fluorometer (Thermo Fisher).

### Whole-exome sequencing

For each subject, indexed, paired-end sequencing libraries were prepared in the GUMC Genomics and Epigenomics Shared Resource from 1.0 ug DNA derived from both the tumor tissue and paired adjacent non-tumor tissue using the Agilent SureSelectXT Human All Exon V6 + COSMIC kit (Agilent, Inc). This platform is designed to generate sequencing libraries covering a target size of 66 Mb enriched for protein-coding regions with enhanced coverage of DNA regions with known somatic mutations identified and cataloged in both The Cancer Genome Atlas (TCGA Research Network: http://cancergenome.nih.gov/) and Catalogue of Somatic Mutations in Cancer (COSMIC) databases^60^. Sequencing was performed on the Illumina HiSeq. 4000 platform using paired-end 150 bp reads to an average depth of coverage of 780x for tumor samples and 690x for paired adjacent non-tumor samples.

### Sequence data analysis

Paired-end, interleaved raw DNA sequence reads in FASTQ format were combined, annotated, and aligned to the human reference genome b37 + 36 Mb decoy contig sequences (http://www.ensembl.org/) using BWA-mem^61^. The resulting mapped sequence SAM files were converted to BAM files using the Picard package (http://broadinstitute.github.io/picard) to clean up mapping artifacts and to identify sequence reads that result from duplicated library fragments. The BAM file alignments were additionally processed using GATK^62^ to correct alignment errors and artifacts that arise due to indels, and perform base quality score recalibration (BQSR). Tumor and adjacent non-tumor pairs were also co-realigned for somatic variant analysis to ensure that the indel positions are consistent between the matched samples.

### Identification of tumor-specific somatic mutations

After applying these stringent filtering criteria, analysis-ready BAM files were processed for analysis of tumor and adjacent non-tumor samples to call raw variants and display SNPs and indels relative to the reference genome using GATK HaplotypeCaller^62^. The resulting VCF call sets were then refined to reduce the number of false positives using variant quality score recalibration (VQSR). Low confidence and likely false positive variants were filtered by applying thresholds to mapping quality, normalized variant quality, and other variant statistics that indicate the presence of strand bias, differences in mapping qualities, and position of ref/alt bases in reads supporting the reference and alternate alleles.

High confidence VCF call sets from each tumor and matched adjacent non-tumor sample were analyzed concurrently to detect somatic tumor-specific single nucleotide substitutions and small indels (<100 bp) using a combination of MuTect^63^ and VarScan^64^. MuTect and VarScan both apply several variant filters to detect false positive SNVs generated due to mapping and sequencing errors. A variant was identified as a false positive if it was observed in the matched normal sample, existed near a proximal gap, exhibited poor mapping or strand bias, was triallelic, or if alternate alleles were clustered at a consistent distance from the sequence read ends. Allele frequency thresholds were applied to variants that were classified as somatic. If the variant allele frequency was >5% in tumor and <5% in normal, it was marked as high confidence. In addition, COSMIC (cancer.sanger.ac.uk) and dbSNP Build ID: 137 (http://www.ncbi.nlm.nih.gov/SNP/) databases were used, respectively, to keep or remove somatic SNVs and obtain a high confidence set.

ANNOVAR^65^ was used to functionally annotate variants with allele frequencies in the 1000 Genomes Project, NHLBI-ESP 6500 exomes, CLINVAR, COSMIC, NCI60 databases. Any variants annotated in the 1000 Genomes Project with an alternate allele frequency >1% were filtered out as common variants. To investigate whether the observed somatic mutations were likely to result in phenotypic changes in amino acid function, we applied 3 commonly used predictors of nonsynonymous mutations on protein function, namely SIFT^66^, PolyPhen-2^67^, and the Ensembl Variant Effect Predictor^68^. All of the procedures described above were performed using standard parameters according to GATK Best Practices recommendations^69^.

MutSigCV^70^ was used to identify genes that are mutated more often than expected by chance alone. This approach considers sample-specific factors, which include mutation frequency and mutational spectrum (e.g., the percentages of mutations that are transitions, transversions, and/or nonsense), and genomic position-based factors, such as gene expression levels, replication times, and chromatin state estimation.

### Evaluation of mutation calling

Evaluation of mutation calling was performed using Sanger capillary electrophoresis sequencing to confirm variants discovered by next-generation sequencing. We randomly chose 25 candidate variants and subsequently designed 25 M13-tailed primer pairs using the web-based ThermoFisher Primer Design Tool and genomic coordinates of the variants (GRCh37 (hg19) human genome assembly; https://www.thermofisher.com/us/en/home/life-science/sequencing/sanger-sequencing/pre-designed-primers-pcr-sanger-sequencing.html). PCR was performed using the BigDye Direct Cycle Sequencing Kit (ThermoFisher) using control DNA (CEPH 1347-02). Capillary sequencing was performed on an Applied Biosystems 3730 DNA Analyzer (ThermoFisher). For all 25 primer pair tested, PCR products were obtained using the high-molecular weight control DNA as template. PCR amplification was successful for only 5/25 (20%) of the primer pairs tested when using FFPE-derived genomic DNA as template, possibly due to the highly fragmented nature of the FFPE DNA, which precludes efficient PCR amplification of products >150 bp. Of these, 5/5 (100%) of NGS variants were validated by Sanger data (Supplemental Fig. 1).

### Mutation signature analysis

Variants passing default detection thresholds were combined by sample and converted to MAF format. MAF format variant calls were analyzed in R using the SomaticSignatures package^71^. Non-negative matrix factorization (NMF) was applied to the trinucleotide-context mutation spectra of the seven tumors in this cohort to extract four stable and reproducible mutational signatures. Signatures were compared to the 30 signatures from the Catalogue of Somatic Mutations in Cancer (COSMIC) (http://cancer.sanger.ac.uk/cosmic/signatures) via visual inspection.

### Pathway analysis

Lists of significantly mutated nonsynonymous genes for each tumor type (ASL, CCA, and HCC) were subjected to gene pathway enrichment analysis using the web-based pathways analysis tool Ingenuity Pathway Analysis (IPA). IPA displays the most significant canonical pathways across the entire dataset and the significance values (p = 0.05) for the canonical pathways are calculated by Fisher’s exact test, right-tailed^72^.

### Ethical approval and informed consent

Informed consent for research was obtained by the Russian Human Radiobiological Tissue Repository personnel from all participants and/or their legal guardian/s. The experimental protocols were approved by the Southern Urals Biophysics Institute’s Institutional Review Board. The Georgetown University Institutional Review Board approved an exemption request to study the de-identified materials. All methods were carried out in accordance with relevant guidelines and regulations.

### URLs

TCGA Research Network: http://cancergenome.nih.gov/

ENSEMBL: http://www.ensembl.org/

COSMIC: http://cancer.sanger.ac.uk/

Picard tools: http://broadinstitute.github.io/picard

dbSNP: http://www.ncbi.nlm.nih.gov/SNP/

ThermoFisher Primer Design Tool: https://www.thermofisher.com/us/en/home/life-science/sequencing/sanger-sequencing/pre-designed-primers-pcr-sanger-sequencing.html

## Supplementary information

Supplemental Information for SREP-18-34832A: Somatic mutation signatures in primary liver tumors of workers exposed to ionizing radiation.

## Data Availability

All reported nucleotide sequences have been submitted for inclusion in the National Center for Biotechnology Information (NCBI) repository pending approval, and accession numbers will be inserted into the manuscript at the end of the review process.
