# Supplementary material for: Somatic mutation signatures in primary liver tumors of workers exposed to ionizing radiation
Source: Sci Rep. 2019 Dec 3;9:18199. doi: 10.1038/s41598-019-54773-z (PMC6890664; doi:10.1038/s41598-019-54773-z)

Supplemental Information for SREP-18-34832A: “Somatic mutation signatures in primary liver tumors of workers exposed to ionizing radiation”

David S. Goerlitz[1], Jan Blancato[1], Archana Ramesh[1], Md Islam[1], Garrett T. Graham[1], Valentina Revina[2], Bhaskar Kallakury[1], Jay Zeck[3], Evgeniya Kirillova[2], and Christopher A. Loffredo[1*]

[1] Lombardi Comprehensive Cancer Center, Georgetown University, Washington, District of Columbia, USA

[2] Russian Radiobiology Human Tissue Repository, Southern Urals Biophysics Institute, Ozyorsk, Chelyabinsk Oblast, Russian Federation

[3]Department of Pathology, Georgetown University, Washington, District of Columbia, USA

*Correspondence to cal9@georgetown.edu

Suplemental Table 1. Summary characteristics of the study cohort.

Sample ID	Diagnosis	Accumulated dose due to external exposure (Gy)	Absorbed dose for liver (Gy)	Pu body burden (kBq)	Absorbed dose for bone marrow due to Pu (Gy)	Absorbed dose for liver due to Pu (Gy)	SEX	AGE	Smoking	Smoking interval (year)	Alcohol consumption	Occupation	% Tumor	% Benign	Grade	% Necrosis
116549	ASL	2.2	1.9	1.1	3.6	2.5	M	54	Yes	36	Yes	Metalworker	40%	60%	LOW	0%
116551	ASL	1.0	0.9	32.9	1.3	3.7	F	69	No	-	No	Engineer-	20%	80%	LOW	30%
116583	CCA	2.0	1.7	2.2	4.4	3.1	M	65	Yes	45	Yes	Electrician	60%	40%	1	0%
116595	CCA	2.4	-	-	-	-	M	59	Yes	-	Yes	Builder	75%	25%	2	20%
116577	HCC	2.8	2.5	99.2	-	-	M	51	No	-	Yes	Engineer	70%	30%	3	30%
116565	HCC	0.8	0.7	2.9	2.1	2.1	M	62	Yes	42	Yes	Operator	70%	30%	4	10%
116569	HCC	0.8	-	-	-	-	M	54	No	-	Yes	Builder	60%	40%	4	10%

Suplemental Table 2. Summary of somatic alterations by mutation class.

	ASL_116549 (n=402)	ASL_116551 (n=752)	CCA_116583 (n=238)	CCA_116595 (n=1392)	HCC_116565 (n=2071)	HCC_116569 (n=1715)	HCC_116577 (n=671)
Mutation class	% (No.)	% (No.)	% (No.)	% (No.)	% (No.)	% (No.)	% (No.)
C-T	38.8% (156)	28.7% (216)	29.0% (69)	33.8% (470)	37.4% (775)	34.2% (586)	32.0% (215)
A-G	44.5% (179)	23.4% (176)	36.1% (86)	27.3% (380)	30.7% (636)	27.1% (465)	25.0% (168)
A-C	5.7% (23)	10.4% (78)	10.1% (24)	7.4% (103)	5.8% (120)	7.1% (121)	8.0% (54)
G-T	3.7% (15)	11.7% (88)	9.2% (22)	9.7% (135)	5.8% (120)	7.5% (129)	9.7% (65)
A-T	3.0% (12)	11.3% (85)	1.7% (4)	6.5% (91)	5.6% (117)	5.6% (96)	6.0% (40)
G-C	4.0% (16)	3.1% (23)	10.5% (25)	3.2% (44)	3.4% (70)	3.9% (67)	4.0% (27)
insertion	0.0% (0)	2.7% (20)	1.3% (3)	5.3% (74)	3.8% (78)	4.2% (72)	6.9% (46)
deletion	0.2% (1)	8.9% (67)	2.1% (5)	6.8% (94)	7.5% (155)	10.4% (179)	8.3% (56)

Suplemental Table 3. Summary of somatic alterations in individual tumors by translation impact.

Subject ID (No. of mutations)	ASL_116549 (n=402)	ASL_116551 (n=752)	CCA_116583 (n=238)	CCA_116595 (n=1392)	HCC_116565 (n=2071)	HCC_116569 (n=1715)	HCC_116577 (n=671)
non-silent mutations/Mb	0.4	3.7	1.0	8.4	13.0	9.8	4.1
Translation impact	% (No.)	% (No.)	% (No.)	% (No.)	% (No.)	% (No.)	% (No.)
Non-silent	6.0% (24)	32.4% (244)	28.2% (67)	39.8% (554)	41.4% (857)	37.6% (645)	40.5% (272)
missense	5.3% (21)	17.3% (130)	24.4% (58)	26.6% (370)	29.7% (616)	22.7% (390)	25.2% (169)
nonsense	0.0% (0)	2.0% (15)	0.0% (0)	2.4% (34)	2.3% (47)	2.1% (36)	2.4% (16)
frameshift indel	0.0% (0)	2.1% (16)	0.4% (1)	4.5% (62)	3.4% (71)	2.9% (50)	5.1% (34)
inframe indel	0.2% (1)	0.9% (7)	0.8% (2)	1.9% (26)	2.1% (44)	2.4% (42)	3.7% (25)
splice	0.5% (2)	10.1% (76)	2.5% (6)	4.5% (62)	3.8% (79)	7.4% (127)	4.2% (28)
Silent	94.0% (378)	67.6% (508)	71.8% (171)	60.2% (838)	58.6% (1214)	62.4% (1070)	59.5% (399)
intronic	79.1% (318)	45.3% (341)	29.4% (70)	36.4% (507)	32.1% (665)	36.9% (632)	37.7% (253)
silent	2.0% (8)	8.4% (63)	27.3% (65)	14.6% (203)	16.6% (343)	14.1% (241)	12.8% (86)
UTR	9.2% (37)	9.4% (71)	7.1% (17)	6.3% (88)	6.8% (140)	6.5% (112)	5.4% (36)
ncRNA	1.5% (6)	3.9% (29)	5.9% (14)	2.5% (35)	2.8% (57)	4.7% (80)	3.4% (23)
intergenic	2.2% (9)	0.5% (4)	2.1% (5)	0.4% (5)	0.4% (9)	0.3% (5)	0.1% (1)

Supplemental Table 4. Singleton mutations of interest.

Gene	Protein	Biological function	Tumor type	Reference
FLT3	Fms-like Tyrosine Kinase 3	angiogenesis	ASL	
LARP1	La Ribonucleoprotein Domain Family Member 1	PIK3CA/AKT/mTOR	ASL	40
YWHAE	Tyrosine 3-Monooxygenase/Tryptophan 5-Monooxygenase Activation Protein Epsilon	AKT signaling	ASL	
PBRM1	Polybromo 1	chromatin remodeling	CCA	38, 43, 45
APC	APC, WNT Signaling Pathway Regulator	Wnt signaling	HCC	
BCOR	BCL6 Corepressor	chromatin remodeling	HCC	38

Supplemental Figure 1. Sanger sequencing validation. Mutational analysis was performed on genomic DNA extracted from FFPE tumor samples. A. PAGE1, 304A>C, TTA>TTC, subject ASL_116549; B. AKAP9, 1148C>delC, TCC>TC-, subject HCC_116565; C. AKAP9, 4603C>T, ATC>ATT, subject CCA_116595; D. BRCA1, 384C>A, CCC>CCA, subject ASL_116551; E. TSC1, 2711G>T, AGG>AGT, ASL_116551.

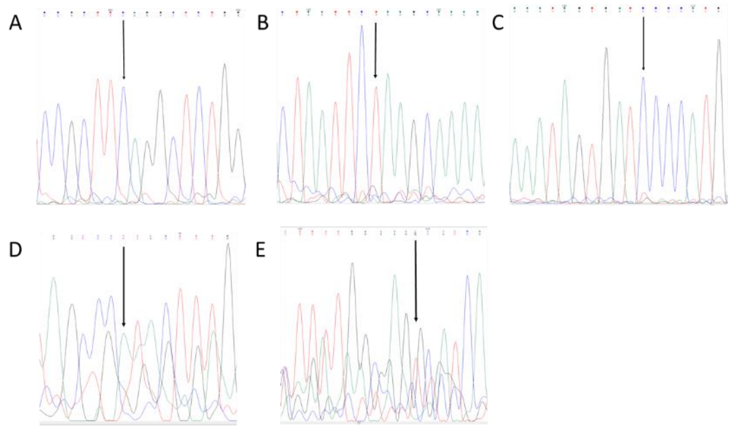

Supplement: Supplementary file 1 — Supplemental Information for SREP-18-34832A: Somatic mutation signatures in primary liver tumors of workers exposed to ionizing radiation. [file 41598_2019_54773_MOESM1_ESM.pdf]
